# Timing and synchrony of birth in Eurasian lynx across Europe

**DOI:** 10.1002/ece3.9147

**Published:** 2022-07-31

**Authors:** Jenny Mattisson, John D. C. Linnell, Ole Anders, Elisa Belotti, Christine Breitenmoser‐Würsten, Ludek Bufka, Christian Fuxjäger, Marco Heurich, Gjorge Ivanov, Włodzimierz Jędrzejewski, Radio Kont, Rafał Kowalczyk, Miha Krofel, Dime Melovski, Deniz Mengüllüoğlu, Tomma Lilli Middelhoff, Anja Molinari‐Jobin, John Odden, Jānis Ozoliņš, Henryk Okarma, Jens Persson, Krzysztof Schmidt, Kristina Vogt, Fridolin Zimmermann, Henrik Andrén

**Affiliations:** ^1^ Norwegian Institute for Nature Research Trondheim Norway; ^2^ Department of Forestry and Wildlife Management Inland Norway University of Applied Sciences Koppang Norway; ^3^ Harz National Park Wernigerode Germany; ^4^ Department of Research and Nature Protection Šumava National Park Administration Kašperské Hory Czech Republic; ^5^ Faculty of Forestry and Wood Sciences Czech University of Life Sciences Prague Prague Czech Republic; ^6^ Foundation KORA Ittigen Switzerland; ^7^ NP OÖ Kalkalpen GesmbH Molln Austria; ^8^ Chair of Wildlife Ecology and Management, Faculty of Environment and Natural Resources University of Freiburg Freiburg Germany; ^9^ Department of Visitor Management and National Park Monitoring Bavarian Forest National Park Grafenau Germany; ^10^ Geonatura Zagreb Croatia; ^11^ Mammal Research Institute Polish Academy of Sciences Białowieża Poland; ^12^ Centro de Ecología Instituto Venezolano de Investigaciones Científicas (IVIC) Caracas Venezuela; ^13^ Department of Zoology, Institute of Ecology and Earth Sciences University of Tartu Tartu Estonia; ^14^ Department of Forestry and Renewable Forest Resources, Biotechnical Faculty University of Ljubljana Ljubljana Slovenia; ^15^ Wildlife Sciences Georg‐August University Goettingen Germany; ^16^ Macedonian Ecological Society Skopje Macedonia; ^17^ Ekoakademi Ekolojik Danışmanlık Aydın Turkey; ^18^ Progetto Lince Italia Tarvisio Italy; ^19^ Norwegian Institute for Nature Research Oslo Norway; ^20^ Latvian State Forest Research Institute “Silava” Salaspils Latvia; ^21^ Institute of Nature Conservation Polish Academy of Sciences Kraków Poland; ^22^ Grimsö Wildlife Research Station, Department of Ecology Swedish University of Agricultural Sciences Sweden

**Keywords:** carnivore, demography, *Lynx lynx*, reproductive phenology

## Abstract

The ecology and evolution of reproductive timing and synchrony have been a topic of great interest in evolutionary ecology for decades. Originally motivated by questions related to behavioral and reproductive adaptation to environmental conditions, the topic has acquired new relevance in the face of climate change. However, there has been relatively little research on reproductive phenology in mammalian carnivores. The Eurasian lynx (*Lynx lynx*) occurs across the Eurasian continent, covering three of the four main climate regions of the world. Thus, their distribution includes a large variation in climatic conditions, making it an ideal species to explore reproductive phenology. Here, we used data on multiple reproductive events from 169 lynx females across Europe. Mean birth date was May 28 (April 23 to July 1), but was ~10 days later in northern Europe than in central and southern Europe. Birth dates were relatively synchronized across Europe, but more so in the north than in the south. Timing of birth was delayed by colder May temperatures. Severe and cold weather may affect neonatal survival via hypothermia and avoiding inclement weather early in the season may select against early births, especially at northern latitudes. Overall, only about half of the kittens born survived until onset of winter but whether kittens were born relatively late or early did not affect kitten survival. Lynx are strict seasonal breeders but still show a degree of flexibility to adapt the timing of birth to surrounding environmental conditions. We argue that lynx give birth later when exposed to colder spring temperatures and have more synchronized births when the window of favorable conditions for raising kittens is shorter. This suggests that lynx are well adapted to different environmental conditions, from dry and warm climates to alpine, boreal, and arctic climates. This variation in reproductive timing will be favorable in times of climate change, as organisms with high plasticity are more likely to adjust to new environmental conditions.

## Graphical abstract

The Eurasian lynx (Lynx lynx) covers three of the four main climate regions of the world, making it an ideal species to explore reproductive phenology in a carnivore. Mean birth date was May 28 (April 23 to July 1), but was ~10 days later in northern Europe than in central and southern Europe. Timing of birth was delayed by colder May temperatures, and we argue that lynx give birth later when exposed to colder spring temperatures and have more synchronized births when the window of favorable conditions for raising kittens is shorter.

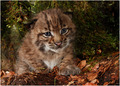

## 1 INTRODUCTION

The ecology and evolution of reproductive timing and synchrony of birth have been a topic of great interest in evolutionary ecology for decades (Chmura et al., 2019; Ims, 1990). Originally motivated by questions related to behavioral and reproductive adaptation to environmental conditions, the topic has acquired new relevance in the face of climate change (Thackeray et al., 2016). Climate change will expose organisms to new environmental conditions, and organisms with a high variation in reproductive plasticity may be more likely to adjust to new conditions (Zettlemoyer & Peterson, 2021). Among large mammals, most of the research focus has been on wild herbivores (English et al., 2012; Pelaez et al., 2020; Rutberg, 1987). The most commonly cited drivers of timing and synchrony are related to timing birth to coincide with favorable forage conditions, avoiding exposing altricial young to predation or inclement weather conditions, and phylogenetic constraints on species reproductive cycles. There is currently evidence for a strong role of environmental seasonality in influencing both timing and synchrony of birth with later births in environments with later peaks in forage and more synchronized births in areas with shorter growing seasons and with less intra‐annual variation (English et al., 2012; Pelaez et al., 2020). There is also evidence that both predation pressure and weather select for synchrony through their influence on neonatal survival (Clutton‐Brock et al., 1987; Michel et al., 2020). For the latter it is expected that individuals will acquire fitness benefits if they can time their reproduction to coincide with resource peaks as lactation is energetically demanding, and higher density of resources can translate into smaller foraging radii, and therefore, less time away from vulnerable young (Boutin & Lane, 2014; Thackeray et al., 2010, 2016).

Secondary consumers are less sensitive to climate change than primary consumers (Thackeray et al., 2016). In ungulates there is a clear fit between timing of birth and high‐quality forage, i.e., resource peak (Boyce, 1979; Pelaez et al., 2020). However, for carnivores, the pulsed availability of resources is more complex. Anti‐predator behavior of neonatal prey may imply that there is a lag between when prey are born and when they are vulnerable to predation (Linnell et al., 1995). Herbivore prey can also be in poor condition after dry and hot summers (Gaillard et al., 1997; Vucetich et al., 2005), or after cold and snow‐rich winters (Grøtan et al., 2005), which makes them more vulnerable to predation. Moreover, prey species can also be migratory, implying that carnivores can be affected by both temporal and spatial variability making a continuous food supply of the young challenging (Walton et al., 2017). All of these suggest a malleable and lagged relationship between carnivore birth pulses and those of their prey.

Reproductive biology is well known in many mammalian carnivores; however, in contrast to herbivores, there has been relatively little research on reproductive phenology and the factors driving timing and synchrony of birth in mammalian carnivores (but see: Garcia‐Rodriguez et al., 2020; Mahoney et al., 2020). Prevailing hypotheses suggest that the timing and synchrony of birth in carnivores are driven by a need to protect altricial young from inclement weather, especially for the species that do not use excavated dens (Boutros et al., 2007), and by seasonality in access to prey at times of the year when energetic needs are greatest (Gittleman & Thompson, 1988).

Most felid species reproduce year‐around (Andrews et al., 2019), although there is often some observed seasonal cyclicity, especially for species living in more temperate climates. Also, within the same species, timing of birth can vary depending on local weather conditions. As an example, both pumas (*Puma concolor*) and leopards (*Panthera pardus*) demonstrate highly variable climate‐dependent birth peaks but with births occurring in all months of the year (Balme et al., 2013; Laundré & Hernandez, 2007). The wide variation in timing of birth in puma is also attributed to the fact that, irrespective of season, pumas mate soon after the female has separated from the kittens, either due to death of the kittens or dispersal (Jansen & Jenks, 2012; Laundré & Hernandez, 2007). Thus, while a female may time the first birth to the period when temperatures are mild and prey are readily available, subsequent births will simply occur a certain number of months after she regains independence from kittens. In contrast, most species of the lynx genus follow a unique reproductive strategy with a monoestrus cycle where ovulation generally occurs once a year (Jewgenow et al., 2014; Painer, Goeritz, et al., 2014; Painer, Jewgenow, et al., 2014). This should lead to a greater degree of birth synchrony in lynx species than in most other felids. In addition, lynx kittens become independent before their first birthday (Samelius et al., 2012; Zimmermann et al., 2005), which permits females to fit a complete reproductive cycle into an annual cycle. However, no study has yet explored the variation in reproductive phenology of any lynx species and the mechanism driving timing and synchrony of birth. This is important because phenological plasticity can play an important role in shaping species' responses to ongoing climate change.

The Eurasian lynx (*Lynx lynx*), the largest of the lynx species, occurs across the Eurasian continent, covering three of four main climate regions of the world (arctic, temperate, and subtropical). Thus, their distribution includes a large variation in climatic conditions, being colder and more seasonal further north, making it an ideal species to explore patterns of variation in timing and synchrony of birth over a large spatial scale. The general timing of the reproductive cycle of the Eurasian lynx (hereafter lynx), i.e., mating in late winter/early spring and giving birth in late spring/early summer after a gestation period of 66–70 days (Painer, Jewgenow, et al., 2014), is likely an adaptation to long‐term climate patterns to ensure the most favorable conditions to bear and rear young (Bowyer et al., 1998). However, there may still be some flexibility within this cycle allowing for local adaptation in timing and synchrony of births. From first principles we would expect that the major constraint is related to avoiding exposing altricial neonates to inclement weather, a factor that increases in severity with latitude and elevation, but also timing birth to best fit temporal patterns in resource distribution to enable this income breeding species (Jönsson, 1997) to best satisfy its energetic needs. As lynx use dens relatively exposed to external conditions (Boutros et al., 2007; Schmidt, 1998), we expect that lynx at northern latitudes will have a later timing and a shorter birth period due to more extreme climates and later onset of spring, than lynx at more southern latitudes (Hodge et al., 2011). Thus, there will be a stronger stabilizing selection pressure toward giving birth at the optimal time in northern latitudes. Kittens born too early may experience temperatures cold enough to be lethal, but because of the short growing season, timing also needs to be optimized to the development of the kittens. Kittens born too late may experience difficulties if they are too small when winter sets in.

**FIGURE 1:**
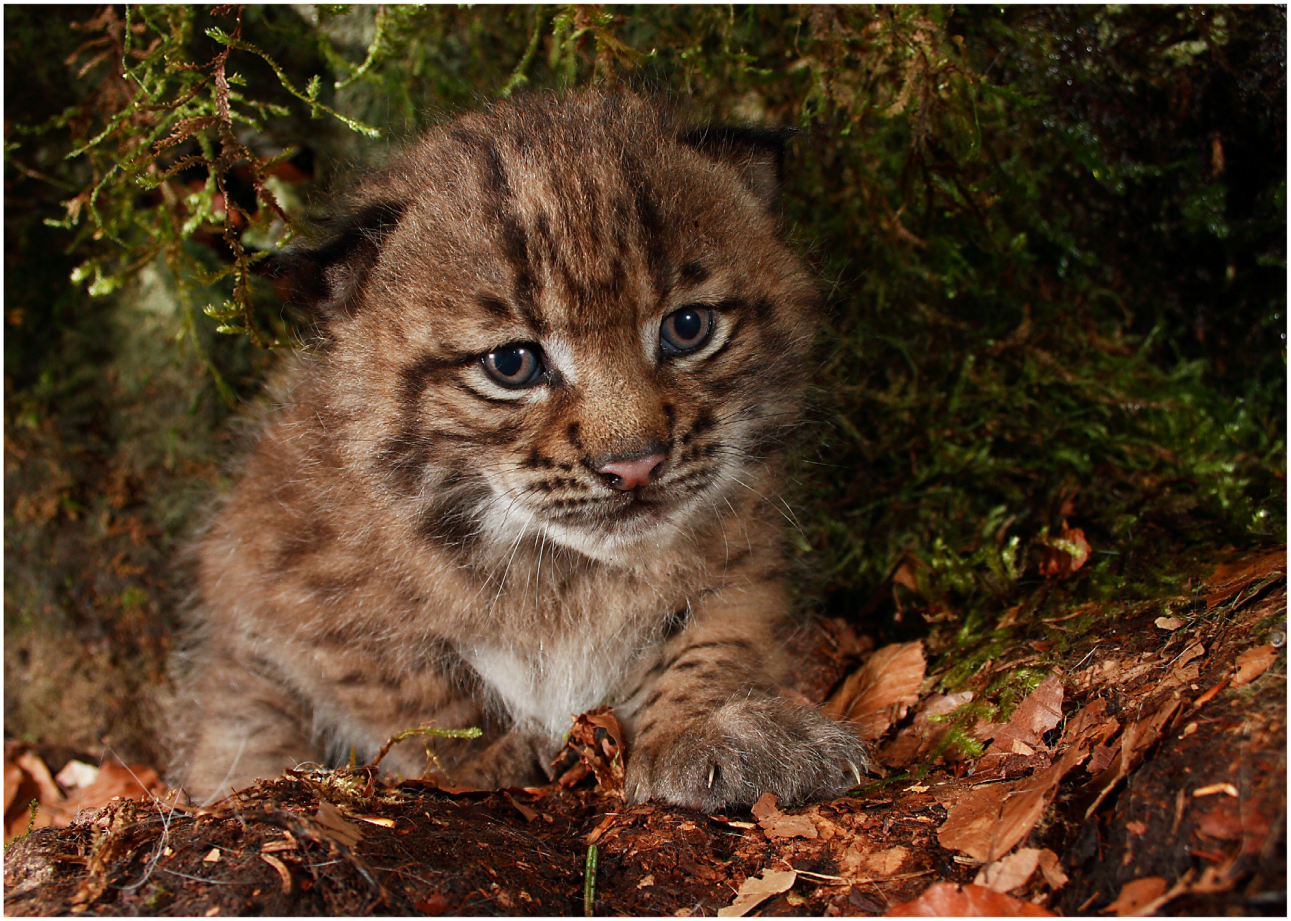
Lynx kitten in Slovenia on July 4th. Photo: Miha Krofel

To test for this, we used data from lynx reproduction events (Figure 1) across most of the lynx distribution in Europe, with lynx birth locations ranging from 40°N to 70°N. As latitude may be a poor proxy of climate in mountainous or coastal regions, where small displacements in elevation or distance from coast can result in nonlinear temperature changes (Loarie et al., 2009), we also used location‐specific mean temperature at the time of parturition and elevation to assess which of these interconnected variables best predict birth date in lynx. In addition, we tested if the timing of birth affected the survival of the kittens. If there is a strong stabilizing selection to give birth at the optimal time, survival will be lower for kittens born early or late, i.e., kitten survival will be affected by the deviation from mean birth date (English et al., 2012; Rode et al., 2018).

## 2 MATERIALS AND METHODS

### 2.1 Reproductive data

**FIGURE 2:**
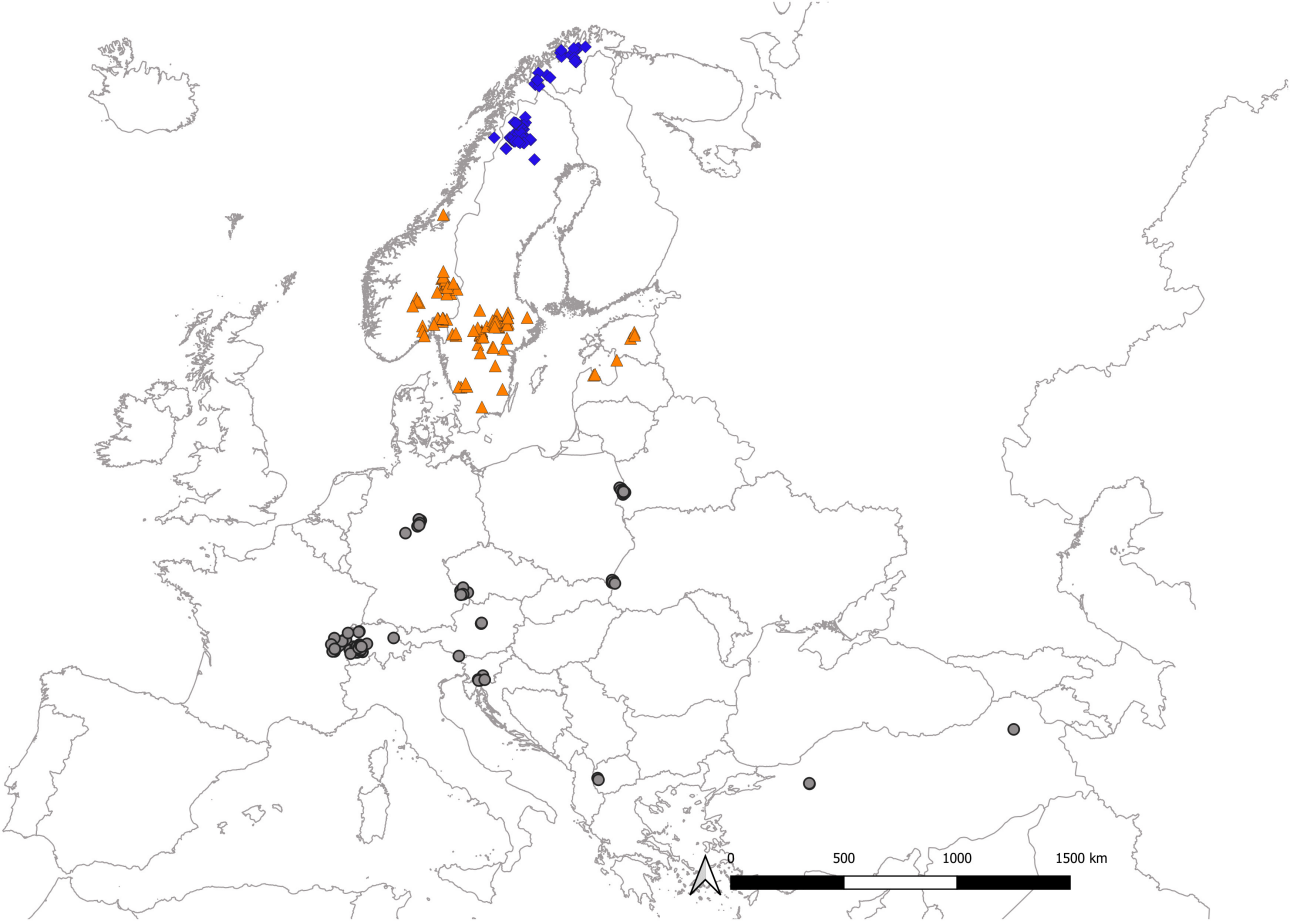
Documented lynx reproduction events (*N* = 313) across Europe 1988–2021. Reproductive events were classified into three geographical regions based on latitude; >65°N (blue diamonds), 65–55°N (orange triangles), and <55°N (gray dots).

Data on reproduction were available for 313 reproductive events by 169 female lynx monitored across Europe and the Asian part of Turkey (hereafter addressed as “Europe”) between 1988 and 2021 (Figure 2). Data were provided by members of the Eurolynx network (https://www.eurolynx.org; Heurich et al., 2021) and originate from 15 countries (Sweden, Norway, Switzerland, Germany, Poland, Czech Republic, France, Austria, Estonia, Slovenia, Latvia, Turkey, North Macedonia, Italy, and Croatia).

Female lynx were captured and equipped with VHF‐ or GPS‐collars following different national handling protocols adapted to local environmental conditions and legislation (see Appendix S1 for details). Female lynx adopt a central‐place foraging behavior centered on a natal site for the first weeks of the kittens' lives (Breitenmoser et al., 1993; Krofel et al., 2013; Schmidt, 1998). This behavior enables the birth site location, and the parturition date, to be determined by examination of high‐resolution GPS‐location data or intensive monitoring by VHF telemetry. All reproductive events were confirmed by a visit to the natal site when kittens were between 0 and 8 weeks old, or by observing one or more kittens with the female later the same year. We assume that the kittens were born on the first day the female started using the natal birth site location and reduced movements to a minimum. For some individuals without GPS‐collars, the first day of denning could not be determined directly from movement data. Birth date for these individuals was either estimated from a regression model based on weights of other kittens with known birth date (Mattisson et al., 2020) or from the developmental stage of the kittens, estimated during natal site visit. Three reproduction events (from Slovenia, Turkey, and Czech Republic) were found by chance (i.e., females not collared), with one belonging to a known female (later confirmed by camera trap images).

The number of kittens in the litter (litter size) was first recorded during the visit to the natal site. The number of kittens still with the mother at the onset of winter (between October and January) was later determined by snow tracking, camera trapping, or visual observations to estimate survival of the kittens (c.f. Gaillard et al., 2014). It was not always possible to determine the individual identity of those kittens that survived and those that died.

Reproductive events were classified into three geographical regions: >65°N, 65–55°N, and < 55°N (Figure 1). This division was based both on the data distribution and sample size (Figure A1) and broad‐scale environmental representation. Events in the northern region (Northern Scandinavia) were located in the north Alpine environmental zone (The Environmental Stratification of Europe [ed.ac.uk]) representing the highest seasonality and lowest temperatures of the three regions. Here, the lynx's main prey are semi‐domestic reindeer, which migrate between seasonal grazing areas (Mattisson et al., 2011). Events in the central region (Southern Scandinavia and the Baltics) were located in the boreal and nemoral zones where roe deer are the main prey (Gervasi et al., 2014; Odden et al., 2006; Valdmann et al., 2005; Žunna et al., 2011). Events in the southern region (Central and Southern Europe and Turkey) are more dispersed across several environmental zones (Continental, South alpine, Atlantic, Mediterranean, Anatolian) and elevation, but the sample size would be too small to separate further. In this area, lynx experience a high variation in available prey species, where some populations mostly prey on small to medium‐sized ungulates (Belotti et al., 2015; Krofel et al., 2011; Molinari‐Jobin et al., 2007; Schmidt, 2008; Vogt et al., 2018) while others rely on smaller prey (Mengüllüoğlu et al., 2018). Most of the lynx populations occurring in the southern region originate from reintroductions (except for the ones in Poland, North Macedonia, and Turkey), an ongoing process starting in the 1970s, but the genetic origin is mostly from the same region, i.e., the Carpathian Mountains. However, lynx in the Harz mountains in Germany descend from zoo stock, which includes Baltic/Scandinavian, Siberian, and Carpathian origin (Linnell et al., 2009; Mueller et al., 2022). Sample size was relatively even between the regions (>65°N = 101 events, 65–55°N = 109, and <55°N = 103).

### 2.2 Environmental data

We derived average monthly temperature data from WordClim v2.1. climate data (https://www.worldclim.org) for 1970–2000 (Fick & Hijmans, 2017) at a 30‐s resolution (~1 km^2^) for the months where the majority of births occurred (May–June). We are aware that there is a temporal mismatch between this data and data on lynx reproduction, but this was the most recent available dataset from Worldclim on average temperature (accessed Sep. 2021). However, we expect spatial variation between birth sites to be greater than temporal variation between years allowing us to use this dataset. Elevation was derived from the European Digital Elevation Model (EU‐DEM) at a resolution of 25 m (https://land.copernicus.eu).

As we only had temperature on a monthly average over many years, we estimated an index of approximate temperature at the time of birth (i.e., this is not the exact daily temperature) to be able to explore whether lynx give birth at similar temperature ranges across Europe. We did this by creating a linear regression for each birth site location, where the mean value for each month (April–July) was set to represent the 15th of that month. We then used this model to extrapolate between monthly averages and predict a temperature for the date of birth for each reproductive event (using ordinal dates).

### 2.3 Statistical analyses

**FIGURE 3:**
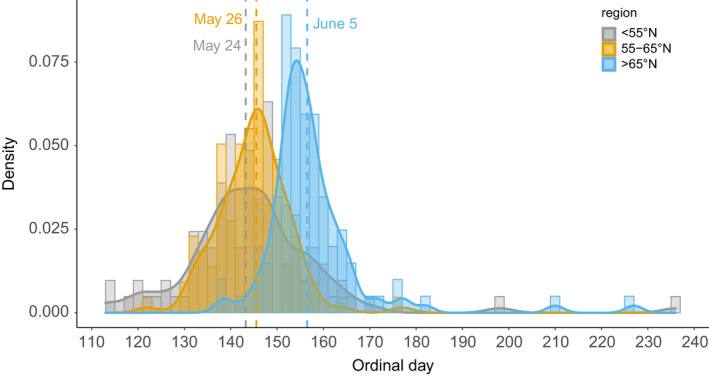
Distribution and density of reproductive events based on birth date represented as ordinal days separated between three different geographical regions; >65°N (blue), 55–65°N (orange), and <55°N (gray) and the vertical striped lines mean birth date for each region. The four birth dates later than ordinal day 190 were considered as outliers and are not included in the mean values, but are included here for illustration.

Birth dates were transformed to ordinal dates (i.e., ranging from 1 to 365/366, where 1 = January 1) to be able to compare across years. As we wanted to assess environmental effects on the timing and synchrony for birth date, we chose to remove four outliers (Figure 3) in the statistical analyses. These were births occurring very late, and even though highlighting the plasticity in timing of birth, these were most likely an effect of lack of mating opportunities or replacement litters rather than an adaptation to environmental conditions. Two events occurred in Scandinavia, likely due to lack of mating opportunities, and are discussed further in Mattisson et al. (2020). Another was an introduced female that was released in Austria on March 25 (i.e., at the end of the mating season). At the time of release, she was not pregnant. The fourth and latest event (Aug 24) from Switzerland was a confirmed replacement litter after losing the previous litter born on May 17. Mean birth date for each geographical region was estimated using a linear mixed model where female lynx ID was included as a random intercept.

To assess if synchrony of birth was impacted by climatic seasonality, we estimated the coefficient of variation (CV) for birth date for each geographical region and tested for differences in CV using a modified signed‐likelihood ratio test (MSLRT; Krishnamoorthy & Lee, 2014) in R‐package *cvequality* (Marwick & Krishnamoorthy, 2019).

To determine if the timing of birth affected survival of the kittens, we used a mixed logistic regression. Survival was estimated from the time of the field control at the birth site (when kittens were 0–8 weeks old) to the period October–January (when kittens were 5–8 months old).To assess whether being born relatively late or early affected survival, we standardized birth date for each geographical region before entering the model. We included standardized birth dates as a quadratic term to allow for a nonlinear response in survival. We additionally included geographical region and litter size as independent variables in the model. Litter size is known to influence lynx kitten survival (Gaillard et al., 2014). Litter ID and female lynx ID were included as random intercepts to account for potential individual differences in general survival.

To identify which environmental variables best explained variation in birth date, we used a linear regression mixed model with birth date as the dependent variable and average temperature in May and June (when the majority of births occur), elevation and latitude as explanatory variables. Female lynx ID was included as a random intercept. As all these independent variables are correlated, with lower temperatures at higher elevation and at higher latitude, we compared each single variable model against each other using AIC. In addition, temperature and elevation were tested for best fit when entered as a linear or a quadratic term.

All statistical analyses were performed using software R (R Core Team, 2020) and the packages *sp* (Bivand et al., 2013), *raster* (Hijmans, 2022), *lme4* (Bates et al., 2015), *ggplot2* (Wickham, 2016), *tidyverse* (Wickham et al., 2019), *plyr* (Wickham, 2011), and *emmeans* (Lenth, 2022).

## 3 RESULTS

### 3.1 Timing and synchrony of birth

Mean birth date was May 28 and ranged from April 22 to July 1 (Figure 3, *N* = 309 after excluding four births between July 17 and August 24). Ninety percent of the births occurred within 1 month (5%–95% quantiles; May 12–June 13). Mean number of kittens in a litter at den visits in summer was 2.2 ± 0.05 SE (1–5, *N* = 279). Mean litter size at onset of winter was 1.2 ± 0.06 SE (0–3, *N* = 261 including all litters with known fate). Litter size at onset of winter among litters with at least one surviving kitten was 1.7 ± 0.05 SE (1–3, *N* = 185).

Mean birth date was significantly later in the northern region (June 5 ± 1.25 SE, *p* < .001), than in the central (May 26 ± 1.04) and the southern region (May 24 ± 1.02, Figure 3). Birth dates were relatively synchronized across Europe (CV = 7%), but more so in the two northern regions (CV = 4.5% at >65°N, CV = 5.2% at 55–65 N°) than in the southern region (CV = 7.9% at <55°N; MSLRT = 34.5, *p* < .001, Figure A2).

Multiple reproductive events were available for 68 lynx females (2–8 events per female). Birth dates of the same females among years varied between 0 and 28 days (mean 10 days ±0.9 SE between the earliest and the latest birth date for each female). In addition, one of the late birth dates that was treated as an outlier (July 17, Figure 3) was from a female with one additional year of known birth date. Her other birth date was on May 20, i.e., 58 days earlier (Figure A3). The one documented replacement litter included in our data (i.e., two birth events in the same year) was born 99 days after the first litter.

**FIGURE 4:**
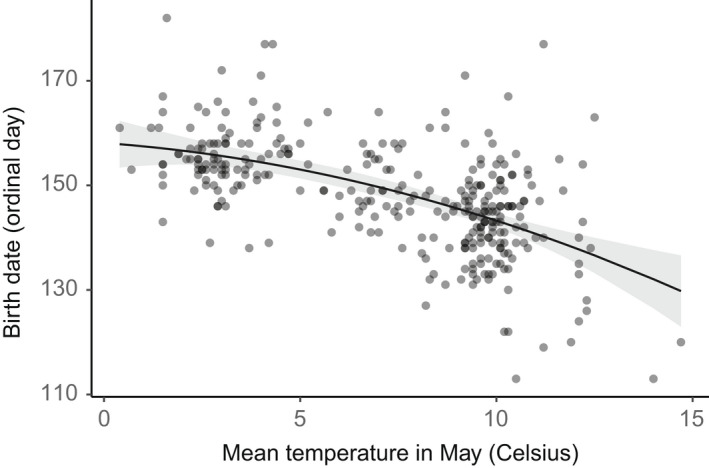
Eurasian lynx birth dates in relation to mean May temperature at 309 birth locations from 167 female lynx. Dots represent raw data (overlapping points give darker color), and the line shows the estimated birth date with 95% confidence intervals from a linear model including female lynx ID as a random intercept.

**FIGURE A1:**
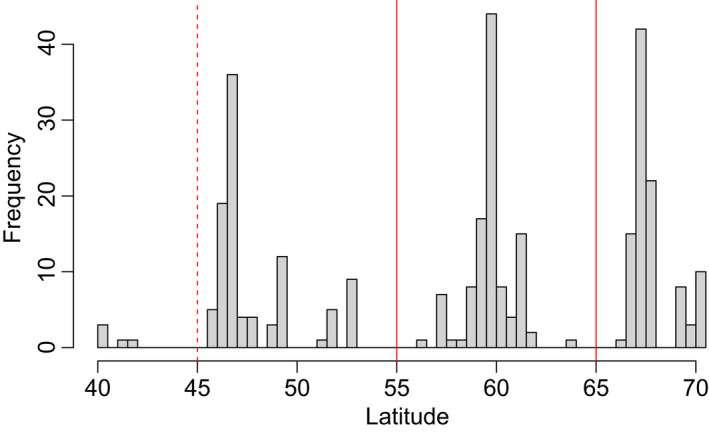
Distribution of reproductive events based on latitude at birth site. Based on this, events were classified into three regions; >65°N, 55–65°N, and <55°N. Data <45°N was too scarce to be separated out.

**FIGURE A2:**
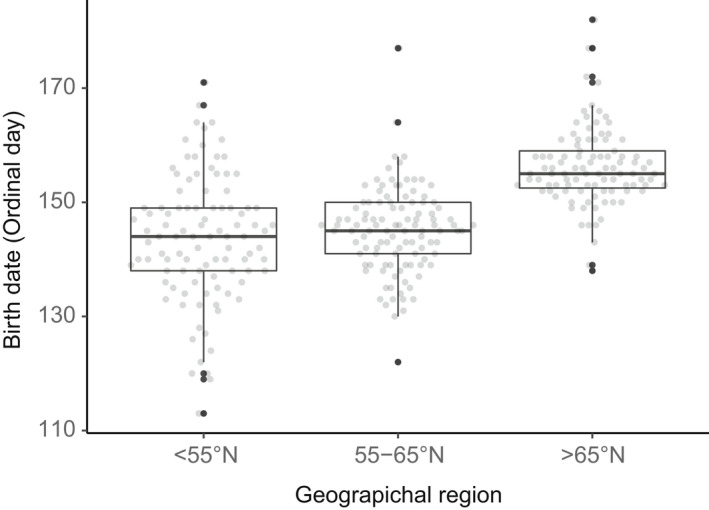
Timing and synchrony in birth dates of lynx in three geographical regions, separated by latitude, across Europe.

Timing of birth was best explained by mean temperature in May as a quadratic term where births occurred later at lower mean temperatures (Figure 4, Table A1, ΔAIC_linear_ = 15). Although this model clearly ranked highest according to AIC, models including June temperature (similar relation as May temperature) and Latitude (births occurred later at higher latitudes) still showed a relatively good model fit (Table A1), while we could not detect any relationship between elevation and timing of birth (Table A1). Temperature, which is a function of latitude and elevation (Figure A5), explained more of the variation in birth date than those variables alone.

Mean temperature, based on estimated temperature at the time of birth, was 10.0°C (± 0.19 SE) in the southern region, 10.8°C (± 0.13 SE) in the mid region and 6.8°C (± 0.15 SE) in the northern region, ranging between 3.2°C and 16°C degrees across Europe.

### 3.2 Kitten survival

Data on survival and litter size at birth were available for 245 litters, totalling 532 kittens. Of these, 289 kittens from 172 litters were still alive at the onset of winter, i.e., 30% of the females lost the complete litter, and 46% of the kittens died, before onset of winter. Whether kittens were born relatively late or early did not affect kitten survival (Figure A4, Table A2). However, kittens from litters of two had a higher probability of surviving than kittens from litters of three (*p* = .01) or four (*p* = .01, Figure A4), based on estimated marginal means. There was weak evidence for lower survival in the northern region >65°N compared with the southern region <55°N (*p* = .09, Figure A4, Table A2). Survival for litters at mean birthdate was 0.54 (0.44–0.63 95% CL).

## 4 DISCUSSION

**FIGURE A3:**
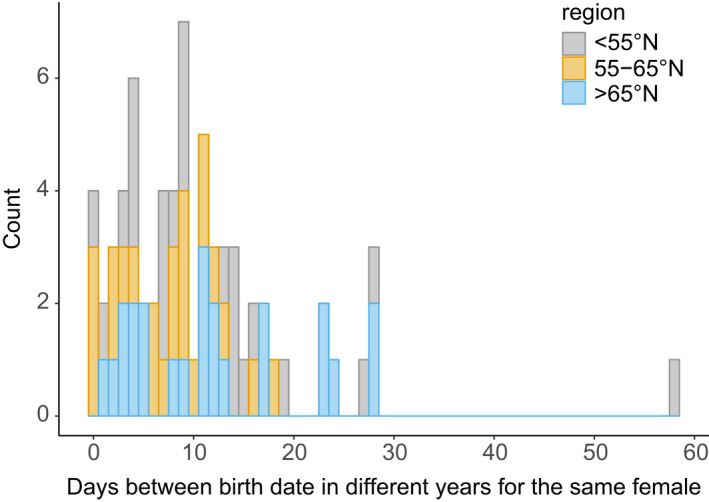
Variation in birth date for individual lynx females among years (i.e., number of days between the earliest and the latest birth date observed for the same female). Counts of observations (*y*‐axis) are stacked and colors represent the geographical region of the female.

**FIGURE A4:**
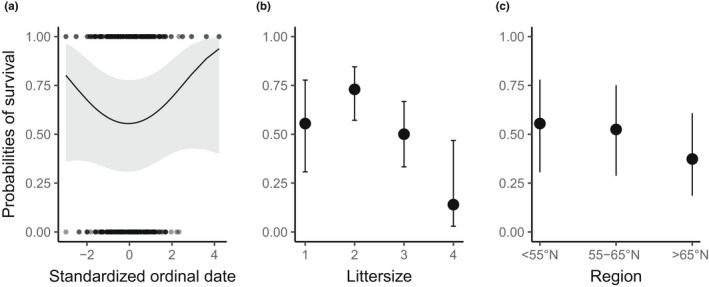
Predicted probabilities (with 95% CI) of survival for lynx kittens depending on (a) timing of birth, (b) litter size, and (c) geographical region. Birth dates (ordinal dates 1–365) were standardized on regions before entering the model and the lowest and highest values represent relatively early and late birth dates in each region. Dots in a) represent raw data. Note that the 95% CI in (a) and (c) are to large to draw any meaningful conclusions.

This study provides a unique broad‐scale analysis on timing of birth in a widely distributed felid species across a large study area spanning 30 degrees of latitude. The results reveal that Eurasian lynx show variation in birth date across its range, with lynx located north of 65° giving birth on average 10–11 days later than lynx located further south. This implies that the unique reproductive system of lynx, where persistent corpora lutea force lynx into a synchronized and monoestrus reproductive system (Painer, Jewgenow, et al., 2014), does allow for some limited flexibility in birth date, both within and among individuals. The variation within individuals (in different years) was actually in the same range as variation among individuals (Figure A3). The variation in timing of birth depending on latitude was mainly driven by mean temperature in spring/early summer where colder temperatures delayed birth. Like Mahoney et al. (2020), we found that local temperature was a more informative driver of phenology in a carnivore than latitude. Even though we had data over two decades, we were unfortunately not able to test if the timing of birth has changed over time, as data were too limited and unevenly distributed in time over the latitude gradient, to draw meaningful conclusions.

**FIGURE A5:**
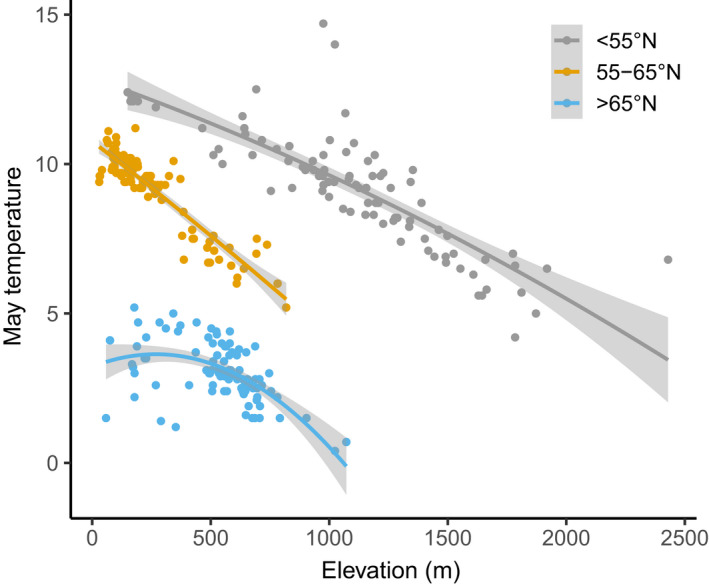
Relationship between mean temperature in May and elevation at birth site locations for Eurasian lynx in three different geographical regions based on latitude; >65°N (blue), 55–65°N (orange), and < 55°N (gray).

Lynx give birth to relatively small and very altricial neonates (Gaillard et al., 2014) often in simple birth lairs that offer little thermal protection (White et al., 2015), although a variety of structures can be used as birth sites (Boutros et al., 2007; Schmidt, 1998). Lynx are income breeders (Jönsson, 1997) and must rely on currently available resources to meet the increased energetic demands of late gestation and early lactation. The female, who alone cares for the kittens, must spend a considerable time away from the birth site hunting already from an early age of the kittens, leaving them exposed. Severe and cold weather may directly affect the neonates via hypothermia and given these facts it would appear logical that early season inclement weather will select against early births, especially at northern latitudes. In contrast to our expectations, we did not detect a decrease in survival of kittens born early, but sample sizes were naturally relatively small for the tails in the distribution of birth dates, and the confidence intervals were too large to make robust conclusions. However, if the strong selection pressure against early birth dates is due to a high risk of death of the kittens already in the first couple of days after birth, our method may not have picked these up if the movement pattern did not indicate that the female had given birth. Thus, there could be an unavoidable sampling bias against early birth dates with low kitten survival. However, lynx in the far north gave birth in mean temperatures 3–4 degrees colder than at latitudes below 65°N (Figure A5), even though they gave birth later. This may indicate that they cannot delay time of birth further without consequences in terms of delayed development of kittens, influencing survival in the following winter.

Synchrony of birth may arise as an adaptive consequence of seasonality in climate or resources, since selection should favor individuals that produce young during advantageous conditions (Riehl, 2018). It is notable that lynx show a high synchrony even though the species is somewhat physically capable of more plasticity, as shown by some occasional late births (treated as outliers in our analysis). This fact makes it even more plausible that climate would be such a strong selective factor that synchrony pays off and later births remain the exception. Lynx birth dates were more synchronized in the two northern regions than in the southern one, which may partly be explained by narrower windows of optimal condition in the regions with higher seasonality. However, another explanation is that the southern region, where birth synchrony was weaker, has a higher variability in elevation and longitude creating a wide range of local temperature regimes and thereby birth dates. Late‐born young are likely to be smaller and may suffer higher rates of mortality than those born earlier in many species (Clutton‐Brock et al., 1987). However, we did not observe lower survival of kittens being born late but as mentioned earlier, data were relatively scarce in the tails of the distribution.

The observed concentration of lynx births in early summer coincides with the main birth seasons of some of the lynx prey species, e.g., roe deer (Pelaez et al., 2020) and chamois (Kourkgy et al., 2016) but not for others, e.g., hares (Mengüllüoğlu et al., 2018) and semi‐domestic reindeer (Mattisson et al., 2011), where the birth peaks occur prior to lynx birth peaks. It should however also be noted that roe deer show greater range‐wide variation in birth date than lynx (Pelaez et al., 2020). While lynx may favor chamois at an early age (Molinari‐Jobin et al., 2004), predation on juveniles of the other species seems to peak later in the summer when anti‐predator measures taken by the prey in the immediate postnatal period are less pronounced (Mattisson et al., 2014; Molinari‐Jobin et al., 2004; Panzacchi et al., 2008). This may imply that lynx birth may be more synchronized with an increase in abundance of easy detectable prey rather than with the pulse of births in the prey. Furthermore, resources availability during winter, when the kittens are still dependent on the mother, may be equally important as in summer. Lynx in the far north, relying on migratory prey that are present in the summer but absent in the winter, show a decreased probability of reproducing and a tendency for lowered kitten survival (Walton et al., 2017) implying that there is a demographic cost associated with low resource availability in winter.

Further, because of the covariation of spring temperature with some of the prey's birth seasons, it is almost impossible to disentangle the relative strengths of neonatal thermal tolerance and prey availability. Although the strict seasonal reproduction cycle of the lynx is most likely an evolutionary adaptation to both climate and resource availability, as observed in, for example, moose (Bowyer et al., 1998), our data suggest that the flexibility within this window is mainly driven by thermal tolerance.

Although the general pattern is related to environmental conditions, we see a plasticity within individuals as well. The same female did not always give birth around the same date but birth date could be displaced by up to a month under normal conditions, and 3 months under exceptional occasions (replacement litter). Ovulations in lynx are in general induced, although they can also occur spontaneously (Painer, Jewgenow, et al., 2014). Therefore, ovulation will be controlled by the presence of a male within the period of potential oestrus. Part of the observed variation in birth date may thus simply be due to the timing of mating opportunities (Krofel et al., 2021; Mattisson et al., 2020).

## 5 CONCLUSION

Lynx are strict seasonal breeders that show a certain, but limited, degree of flexibility to adapt the timing of birth to surrounding environmental conditions. We argue that there is evidence for variation in the timing of birth across the lynx distribution range, which is driven by temperature. Lynx in the far north give birth later due to the colder temperature and will raise their kittens during a shorter window of favorable conditions creating a higher reproductive synchrony. Lynx in central and southern Europe show the widest range in timing of birth, suggesting that lynx are well adapted to different environmental conditions, from dry and warm environments in Turkey to high elevation conditions in the Alps. Our data suggest that the reproductive flexibility of the Eurasian lynx to a wide range of temperatures can give an important advantage to this predator in times of climate change, as expected for species higher up in the food chain (Chmura et al., 2019; Thackeray et al., 2016). This reproductive plasticity not only helps explain the wide distribution of this species, but also suggests that lynx will continue to occupy and influence ecological communities under projected climate scenarios predicted for western Eurasia.

## AUTHOR CONTRIBUTIONS

**Jenny Mattisson:** Conceptualization (equal); data curation (lead); formal analysis (lead); methodology (lead); visualization (lead); writing – original draft (lead); writing – review and editing (lead). **John D. C. Linnell:** Conceptualization (equal); data curation (equal); writing – original draft (supporting); writing – review and editing (supporting). **Ole Anders:** Data curation (equal); writing – review and editing (supporting). **Elisa Belotti:** Data curation (equal); writing – review and editing (supporting). **Christine Breitenmoser‐Würsten:** Data curation (equal); writing – review and editing (supporting). **Ludek Bufka:** Data curation (equal); writing – review and editing (supporting). **Christian Fuxjäger:** Data curation (equal); writing – review and editing (supporting). **Marco Heurich:** Data curation (equal); writing – review and editing (supporting). **Gjorge Ivanov:** Data curation (equal); writing – review and editing (supporting). **Włodzimierz Jędrzejewski:** Data curation (equal); writing – review and editing (supporting). **Radio Kont:** Data curation (equal); writing – review and editing (supporting). **Rafał Kowalczyk:** Data curation (equal); writing – review and editing (supporting). **Miha Krofel:** Data curation (equal); writing – review and editing (supporting). **Dime Melovski:** Data curation (equal); writing – review and editing (supporting). **Deniz Mengüllüoğlu:** Data curation (equal); writing – review and editing (supporting). **Tomma Lilli Middelhoff:** Data curation (equal); writing – review and editing (supporting). **Anja Molinari‐Jobin:** Data curation (equal); writing – review and editing (supporting). **John Odden:** Conceptualization (equal); data curation (equal); writing – review and editing (supporting). **Jānis Ozoliņš:** Data curation (equal); writing – review and editing (supporting). **Henryk Okarma:** Data curation (equal); writing – review and editing (supporting). **Jens Persson:** Data curation (equal); writing – review and editing (supporting). **Krzysztof Schmidt:** Data curation (equal); writing – review and editing (supporting). **Kristina Vogt:** Data curation (equal); writing – review and editing (supporting). **Fridolin Zimmermann:** Data curation (equal); writing – review and editing (supporting). **Henrik Andrén:** Conceptualization (equal); data curation (equal); formal analysis (supporting); writing – original draft (supporting); writing – review and editing (supporting).

## FUNDING INFORMATION

The study was supported by Norwegian Institute for Nature Research (JM), Research Council of Norway (Grant numbers 251112, 281092, and 156810), the Norwegian Directorate for Nature Management, the Nature Protection Division of the County Governor's Office for Innlandet, Viken, Vestfold & Telemark, Trøndelag, Nordland, Troms & Finnmark County, Swedish Environmental Protection Agency (grant numbers F‐56‐09, V‐220‐08), the Swedish Research Council FORMAS (grant numbers 2010‐1007, 2015‐01207), Program GEF‐Biodiversity Protection in the Czech Republic, the Slovenian Research Agency (grants numbers P4‐0059 and N1‐0163), the European Commission (grant number LIFE16 NAT/SL/000634), EU Program Interreg IV (Objective 3 Czech Republic—the Independent State of Bavaria, grant number 18), the Environmental Investment Centre in Estonia, Charity Foundation from Liechtenstein, Haldimann Foundation, Stotzer‐Kästli Foundation, Zigerli‐Hegi Foundation, Zürcher Tierschutz, Temperatio Foundation, Karl Mayer Foundation, Ormella Foundation, Federal Office for Environment in Switzerland, hunting inspectorates of the Cantons of Bern, Fribourg, Vaud, Zürich, St. Gallen, Thurgau, Appenzell Innerrhoden, Appenzell Ausserrhoden, WWF Austria, the German Academic Exchange Service, Rufford Foundation (RSGF 11447‐1) and Turkish General Directorate of Nature Conservation and National Parks, Scientific Research Committee in Poland (grant numbers 6P205 034 05 and 3P04F 019 24), Interreg IIIA Neighborhood Programme Slovenia/Hungary/Croatia 2004–2006. The Balkan lynx study was financially supported by the SCOPES programme (Scientific Cooperation between Eastern Europe and Switzerland) from 2010 until 2012, the People's Trust for Endangered Species (2013–2014), the German Federal Environmental Foundation (DBU) for the period 2015–2018, as well as the MAVA foundation through the Balkan Lynx Recovery Programme in the period from 2006 until the present day.

## CONFLICT OF INTEREST

We have no competing interests.

## Supporting information


Appendix S1

## Data Availability

Data are uploaded to Dryad Digital Repository https://doi.org/10.5061/dryad.xksn02vjk.

## APPENDIX A

**TABLE A1:** Model selection and estimates of timing of birth (*n* = 309) in relation to environmental variables. Female lynx individual (*n* = 168) was included as a random variable. Quadratic terms are estimated using the poly function in R (i.e., using orthogonal polynomials). Marginal *R* ^2^ was estimated for each model using Nakagawa *R* ^2^ (Lüdecke et al., 2021).

Model	ΔAIC	Intercept	*β* (±SE)	*β* ^2^ (±SE)	*R* ^2^
May temperature^2^	0	148 (0.57)	−103 (10.3)	−14.4 (9.2)	.32
May temperature	14.7	161 (1.50)	−1.81 (0.18)		.32
June temperature^2^	15.1	148 (0.60)	−94.2 (10.8)	−9.47 (9.7)	.27
June temperature	28.1	172.1 (2.89)	−2.08 (0.24)		.27
Latitude	44.3	117 (4.70)	0.53 (0.81)		.18
Elevation^2^	64.2	147 (0.77)	2.18 (12.3)	−16.5 (12.2)	.01
Null	75.7				0
Elevation	88.8	147 (1.22)	0.0002 (0.0015)		0

**TABLE A2:** Model estimates of survival probabilities (0,1) in lynx kittens (*n* = 532) in relation to timing of birth (birth date), litter size, and geographical region. Birth dates were standardized on regions before entering the model and lowest and highest values represent relatively early and late birth dates in each region. Litter ID (*n* = 245) nested under female lynx ID (*n* = 125) was included as random intercepts. Quadratic terms are estimated using the poly function in *R* (i.e., using orthogonal polynomials).

Fixed effects	*β* (±SE)	*p*‐Value
Intercept^a^	0.35 (0.51)	.50
Birth date	1.44 (3.42)	.67
Birth date^2^	5.69 (3.66)	.12
Litter size 2^b^	0.77 (0.48)	.10
Litter size 3^b^	−0.22 (0.50)	.66
Litter size 4^b^	−2.04 (0.97)	.04
Region 55°N–65°N^c^	−0.12 (0.38)	.75
Region >65°N^c^	0.74 (0.43)	.08

Random effects	Variance (SD)
Litter ID/Female lynx ID	1.29 (1.14)
Female lynx ID	0.59 (0.77)

^a^ Litter size 1 and Region <55°N are the reference.

^b^ In relation to Litter size 1.

^c^ In relation to Region <55°N.
